# Inferior Frontal Sulcal Hyperintensity on Fluid-Attenuated Inversion Recovery Is Related to Cerebrospinal Fluid Clearance *via* Putative Meningeal Lymphatics

**DOI:** 10.14336/AD.2024.0415

**Published:** 2024-04-15

**Authors:** Ziyu Zhou, Ying Zhou, Wang Ran, Shenqiang Yan, Xiao Zhu, Zhongyu Luo, Huihong Ke, Kemeng Zhang, Mengmeng Fang, Jianzhong Sun, Min Lou

**Affiliations:** ^1^Department of Neurology, Second Affiliated Hospital of Zhejiang University, School of Medicine, Hangzhou, China.; ^2^Department of Radiology, Second Affiliated Hospital of Zhejiang University, School of Medicine, Hangzhou, China.

**Keywords:** Glymphatic System, Cerebrospinal Fluid, Cerebral small vessel diseases, Magnetic Resonance Imaging

## Abstract

Inferior frontal sulcal hyperintensity (IFSH) on FLAIR sequence may indicate elevated cerebrospinal fluid (CSF) wastes. The objective of this study was to investigate its association with the clearance function of putative meningeal lymphatic vessels (mLVs). We included patients who underwent FLAIR sequence and dynamic contrast MRI with intrathecal administration of contrast agent. The visibility of IFSH was quantitatively assessed by measuring the mean signal intensity of inferior frontal sulci on 2D FLAIR. The clearance function of putative mLVs was defined as the percentage change of signal unite ratio in the parasagittal dura from baseline to 4.5, 15 and 39 hours after intrathecal injection on dynamic contrast MRI. Additionally, imaging markers of cerebral small vessel disease, including white matter hyperintensities and enlarged perivascular spaces, were measured. Correlation analysis and linear regression were employed to verify the association of IFSH with the clearance function of mLVs. A total of 76 patients were included in the study. The visibility of IFSH was found to be associated with the percentage change of signal unite ratio in parasagittal dura from baseline to 15 and 39 hours in adjusted analyses. Furthermore, the visibility of IFSH was positively related to the age, scores of both periventricular and deep white matter hyperintensities, and the grade of enlarged perivascular spaces in centrum semiovale. These findings suggest that the visibility of IFSH on 2D FLAIR may serve as an indicator of clearance dysfunction of mLVs and may be implicated in the development of cerebral small vessel disease.

## INTRODUCTION

The cerebrospinal fluid (CSF) typically exhibits a lower signal on FLAIR MRI in comparison to the brain tissue. Consequently, the inferior frontal sulci, which are filled with CSF and lie between the gyrus recti and the olfactory sulci, are theoretically expected to appear hypointense on FLAIR imaging. However, the occurrence of hyperintensity in the inferior frontal sulci, referred to as inferior frontal sulcal hyperintensity (IFSH), is a common phenomenon in elderly subjects, and its underlying cause remains unclear to date [1].

Studies have suggested that an increment of waste products within the CSF, such as protein, blood or cell debris, can elevate the signal intensity of CSF, causing hyperintensity on FLAIR imaging [2, 3]. The glymphatic system is recently discovered to be a cerebral metabolite drainage system [4]. In the previous studies utilizing glymphatic MRI with intrathecal injection, it was confirmed that meningeal lymphatic vessels (mLVs) serve as the primary downstream outflow pathways of the glymphatic system in humans [5, 6]. The clearance function of mLVs has been demonstrated to be crucial for the drainage of waste products [7]. Therefore, the hypothesis posited that mLVs clearance dysfunction may lead to an accumulation of metabolic wastes in the inferior frontal sulci, ultimately contributing to IFSH formation. In addition, recent studies have found the presence of IFSH on 3D T2 FLAIR was associated with increasing age, as well as features of cerebral small vessel disease (CSVD) [1, 8]. However, these studies did not thoroughly investigate the correlation between age, CSVD and the signal intensity of IFSH on 2D T2 FLAIR imaging.

In the present study, we evaluated the clearance function of putative mLVs by analyzing the percentage changes of signal unite ratio in the parasagittal dura (PSD) using dynamic contrast MRI (DCE-MRI) with intrathecal administration of a contrast agent. This method has been widely used in prior human studies that focused on mLVs [5, 6, 9]. Concurrently, we employed quantitative assessment of the mean signal intensity of the inferior frontal sulci on 2D FLAIR to measure the visibility of IFSH. The association between IFSH visibility and the clearance function of putative mLVs was then verified. Furthermore, we delved into the impact of IFSH on both clinical and imaging characteristics. Our investigation encompassed demographics, CSVD imaging markers, and cognitive function. This comprehensive approach was adopted based on the assumption that dysfunction in brain metabolic clearance might play a role in the development of CSVD [10, 11].

## MATERIALS AND METHODS

### Study Subjects

Participants who met the criteria for lumbar puncture and willingly participated were enrolled in the study from April 2018 to December 2021. Criteria for lumbar puncture were guided by disease-specific guidelines and the clinical judgment of physicians. Exclusion criteria included a known history of hypersensitivity reactions to contrast agents, severe allergic reactions in general, evidence of renal dysfunction, and pregnant or breastfeeding. For this secondary analysis, additional exclusion criteria were applied: (1) absence of whole brain 2D FLAIR sequence, and (2) absence of scans of the inferior frontal sulci on the whole brain 2D FLAIR imaging.

Demographics (age, gender) and vascular risk factors (history of drinking and smoking, medically diagnosed hypertension, diabetes, hyperlipidemia) were collected based on inpatient medical records or telephone follow-up. Cognitive function was assessed using the Telephone Montreal Cognitive Assessment (T-MoCA), which evaluates cognitive performance across five domains: attention and calculation, language, abstraction, delayed recall, and orientation. The total score was 22 points with higher scores indicating better cognitive function [12]. The T-MoCA was administered over the phone one month after discharge.

This study, including the administration of intrathecal gadolinium agents, received approval from the Ethics Committee of Second Affiliated hospital, School of medicine, Zhejiang University (Approval Number: YAN-2018-111). All clinical investigations were conducted in accordance with the principles outlined in the Declaration of Helsinki. Informed consent was obtained from all patients and their relatives.

### MRI protocol

All subjects underwent 3.0 T MRI scan (GE) using an 8-channel brain phased array coil. The imaging parameters were: high-resolution FLAIR focused on the sagittal sinus: repetition time = 8,400 milliseconds, echo time = 152 milliseconds, inversion time = 2000 milliseconds, flip angle = 90°, thickness = 3.0 mm, no slice gap, field of view = 18 × 18cm^2^, matrix = 320 × 320 pixels; whole brain FLAIR: repetition time = 10,000 milliseconds, echo time = 150 milliseconds, inversion time = 2500 milliseconds, thickness = 5.0 mm, no slice gap, field of view = 24 × 24 cm^2^, matrix = 256 × 256 pixels; T2 weighted image (T2WI): repetition time = 2500 milliseconds, echo time = 4 milliseconds/100 milliseconds, thickness = 4.0 mm, field of view = 24 ×24 cm^2^, matrix = 512 × 512 pixels; susceptibility weighted imaging (SWI): echo time = 4.5 milliseconds [first echo], inter-echo spacing = 4.5 milliseconds, repetition time = 58 milliseconds, field of view = 24 × 24 cm^2^, matrix size = 256 × 256, flip angle = 20°, slice thickness = 2.0 mm, no slice gap, flow compensation was applied; 3D-T1 weighted imaging (3D-T1): repetition time = 7.3 milliseconds, echo time = 3.0 milliseconds, flip angle = 8°, thickness = 1.0 mm, field of view = 25 × 25 cm^2^ , matrix = 250 × 250 pixels.

### Intrathecal Administration of Gadodiamide

The site of intrathecal injection of the contrast agent is L3-4 or L4-5 lumbar intervertebral space. Intrathecal injection of 1 ml of 0.5 mmol/mL gadodiamide (Omniscan; GE Healthcare) was preceded by verifying the correct position of the syringe tip in subarachnoid space in terms of CSF backflow from the puncture needle [6]. Following needle removal, patients were instructed to rotate themselves around the long axis of the body twice, and then remain in the supine position until 4 hours after intrathecal injection.

### Measurement of the clearance function of putative mLVs

All evaluators remained blind to clinical and other image data. A neurologist (R.W., with 5 years of experience) autonomously placed region of interest (ROI) and conducted image evaluations. To assess intra-observer consistency, the same neurologist reevaluated the images in 20 patients after six months. Another neurologist (Z.Y., with 10 years of experience) independently placed ROI in 20 patients to evaluate inter-observer consistency. Following established protocols from prior studies [6, 13], the clearance function of putative mLVs was assessed through the percentage changes in PSD from baseline to 4.5, 15 and 39 hours after intrathecal administration of a contrast agent on high-resolution FLAIR focused on the sagittal sinus. In each patient, we compared the signal unit ratio in PSD at 4.5 hours, 15 hours, and 39 hours, and defined the time point with highest signal unit ratio as the peak time point of CSF tracer enrichment. Early and late filling of mLVs was defined as the time point with relatively higher signal unit ratio before 39 hours (at 4.5 hours and 15 hours) and at 39 hours, respectively. A comprehensive overview of the evaluation process is provided in Supplemental Methods 1 and Supplementary Figure 1.

### Assessment of the visibility of IFSH

**Figure 1.**
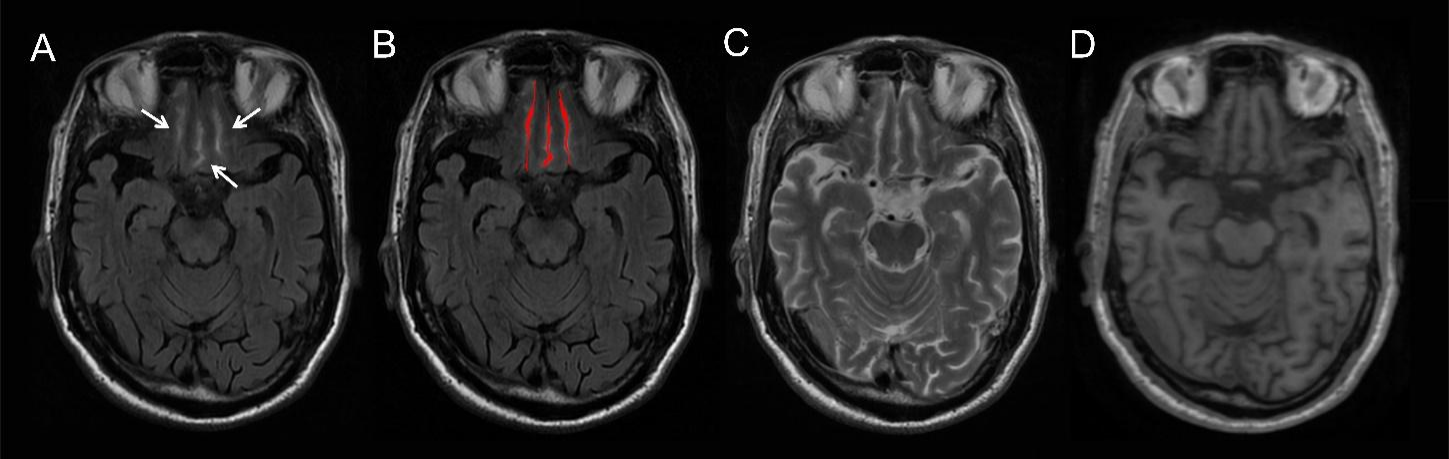
**Representative images of inferior frontal sulcal hyperintensity (IFSH)**. IFSH is defined as the hyperintensities within the inferior frontal sulci on axial whole-brain Fluid-Attenuated Inversion Recovery (FLAIR) images. White arrows depict representative manifestations of IFSH (A). The red region (B) illustrates the placement of the region of interest (ROI), covering the region of the inferior frontal sulci. These hyperintensities should lack corresponding non-cerebrospinal fluid (CSF) signal on T2-weighted imaging (T2WI) (C) and T1-weighted imaging (T1WI) (D).

IFSH was defined as the hyperintensities in the region of the inferior frontal sulci on axial whole brain FLAIR. The quantitative assessment of IFSH visibility utilized the mean signal intensity of the inferior frontal sulci on FLAIR. All evaluators operated with blindness to clinical and other image data. A neurologist (Z.Z., with 4 years of experience) autonomously conducted the measurement using MRIcron© (NeuroImaging Tools & Resources Collaboratory) and repeated the assessment six months later in 20 patients to evaluate intra-observer consistency. Another neurologist (K.H., with 5 years of experience) independently evaluated 20 patients to determine inter-observer consistency. We selected the measurement slice with most prominent IFSH and intact inferior frontal sulci structure, which comprised the central sulcus between the gyrus recti and the olfactory sulci. The ROI drawn on the measurement slice covered the inferior frontal sulci region while avoiding vessels and non-CSF signal region, guided by T1WI and T2WI. Figure 1 illustrates the representative images of IFSH and the placement of the ROI.

### Evaluation of CSVD image markers

Deep and periventricular white matter hyperintensities (WMHs) were visually scored on FLAIR using the Fazekas scale (0-3 for each)[14]. Severe WMHs were defined as grade 3 on periventricular hyperintensities (PVHs) or grade 2 or 3 on deep white matter hyperintensities (DWMHs). Enlarged perivascular spaces (EPVS) were rated according to an established protocol in both the centrum semiovale (CSO) and basal ganglia (BG), with grades ranging from 0 to 4 for each region [15]. A high grade of EPVS was defined as grade 2 to 4, while a low grade of EPVS was defined as grade 0 and 1. Lacunas were defined on FLAIR as 3 to 15 mm diameter cavities with signal intensities similar to CSF in all performed scan sequences across the entire brain [16]. Microbleeds were defined as small, rounded or circular, well-defined low signal lesions within the brain parenchyma, with clear margins ranging from 2 to 10 mm in size on SWI Magnitude images [17]. For detailed information about the evaluation, please refer to Supplemental Methods S2.

### Statistical Analyses

Statistical analyses were performed on SPSS software version 22.0 (IBM Corporation). The normality of data distribution was assessed using the Shapiro-Wilks normality test. Correlations between continuous variables were determined using Pearson correlation analysis for variables that followed a normal distribution, and Spearman correlation analysis for variables did not follow a normal distribution. Differences between continuous variables were determined using independent t-tests. Variables found to be statistically significant in the univariate analysis, or those considered relevant based on previous studies (such as age and sex), were included in the multivariable analyses. Multivariable analyses were performed using linear regression and ordinal logistic regression. Linear regression and ordinal logistic regression model were used to confirm the association between the mean signal intensity of the inferior frontal sulci and CSVD imaging markers after adjusting for age, sex, hypertension and diabetes (included age and sex in model 2; added hypertension and diabetes in model 3). Linear regression model was used to confirm the relationship between the mean signal intensity of the inferior frontal sulci and percentage change of PSD after adjusting age, sex, Fazekas of PVHs, Fazekas of DWMHs and Grade of EPVS in CSO (included age and sex in model 2; added Fazekas of PVHs, Fazekas of DWMHs and Grade of EPVS in CSO in model 3). The Variance Inflation Factor (VIF) was used to test multicollinearity among the covariates within the multicovariate linear regression models. Inter- and intra-observer measurement consistency of the mean signal intensity of the inferior frontal sulci and the percentage changes in signal unit ratio of PSD were analyzed using the interclass correlation coefficient (ICC) in a two-way random and absolute agreement model. Statistical significance was considered at the 0.05 level (two-tailed).

## RESULTS

### Participant characteristics

A total of 76 patients participated in this study, comprising 39 males with a mean age of 56 years (21-85 years) and 37 females with a mean age of 60 years (18-79 years). The final diagnosis included peripheral neuropathy (n = 37), neurodegenerative diseases (n = 16), encephalitis (n = 14), normal pressure hydrocephalus (n = 4), suspected CSF leakage (n = 4), and hepatic encephalopathy (n = 1). The average T-MoCA score was 13.80 ± 4.28. Basic clinical data was presented in Table 1.

**Table 1:** Basic clinical data.

	Total material
**N**	76
**The mean signal intensity of the inferior frontal sulci**	603.38 ± 221.66
**Age (years)**	58.18 ± 14.25
**Female**	37 (48.7%)
**Vascular risk factors**	
**Hypertension**	34 (44.7%)
**Diabetes**	27 (35.5%)
**Hyperlipidemia**	7 (9.2%)
**History of drinking**	18 (23.7%)
**History of smoking**	27 (35.5%)
**CSVD image markers**	
**Fazekas of PVHs**	2 (1, 3)
**Fazekas of DWMHs**	1 (1, 2)
**Presence of cerebral microbleeds**	13 (17.1%)
**Number of microbleeds**	1.3 ± 4.1
**Presence of lacunas**	21 (27.6%)
**Number of lacunas**	1.4 ± 3.0
**Grade of EPVS in basal ganglia**	1 (1, 1)
**Grade of EPVS in centrum semiovale**	2 (1, 3)
**Disease diagnosis**	
**Peripheral neuropathy**	37 (48.7%)
**Encephalitis**	14 (18.4%)
**Normal pressure hydrocephalus**	4 (5.3%)
**Suspected CSF leakage**	4 (5.3%)
**Hepatic encephalopathy**	1 (1.3%)
**Neurodegenerative diseases**	
**Parkinson's disease**	5 (6.6%)
**Motor neuron disease**	3 (3.9%)
**Possible cerebral amyloid angiopathy**	4 (5.3%)
**Multiple sclerosis**	1 (1.3%)
**Multiple system atrophy**	1 (1.3%)
**Lewy body dementia**	1 (1.3%)
**Alzheimer's disease**	1 (1.3%)
**Education years (n = 48)**	7.11 ± 4.14
**T-MoCA total scores (n = 48)**	13.80 ± 4.28
**Attention and calculation**	4.30 ± 1.67
**Language**	1.70 ± 1.09
**Abstraction**	0.61 ± 0.65
**Delayed recall**	1.98 ± 1.81
**Orientation**	5.22 ± 1.17

Abbreviation: CSVD, cerebral small vessel disease; PVHs, periventricular hyperintensities; DWMHs, deep white matter hyperintensities; EPVS, enlarged perivascular spaces; CSF, cerebrospinal fluid; T-MoCA, Telephone Montreal Cognitive Assessment.

### The relationship between the severity of IFSH, demographic information and CSVD image markers

The interclass correlation coefficient for intra-observer and inter-observer consistency in measuring the mean signal intensity of the inferior frontal sulci were 0.952 and 0.907, respectively. The mean signal intensity of the inferior frontal sulci averaged 603.38 ± 221.66. In terms of demographic information, 34 (44.7%) patients had a history of hypertension, 27 (35.5%) had diabetes mellitus, 7 (9.2%) had hyperlipidemia, and 18 (23.7%) and 27 (35.5%) were drinkers and smokers, respectively. Regarding CSVD image markers, for PVHs, 10 (13.2%), 23 (30.3%), 21 (27.6%) and 22 (28.9%) patients were classified as grade 0, 1, 2 and 3, respectively. For DWMHs, 8 (10.5%), 33 (43.4%), 20 (26.3%) and 15 (19.7%) patients were classified as grade 0, 1, 2 and 3, respectively. The median Fazekas score for PVHs and DWMHs were 2 (1, 3) and 1 (1, 2), respectively. Additionally, 21 (27.6%) patients had lacunas, with an average number of 1.4, and 13 (17.1%) patients had cerebral microbleeds, with an average number of 1.3. For EPVS in BG, 6 (7.9%), 56 (73.7%), 6 (7.9%), 7 (9.2%) and 1 (1.3%) were graded as 0, 1, 2, 3 and 4, respectively. In the CSO, 5 (6.6%), 24 (31.6%), 26 (34.2%) and 21 (27.6%) patients were graded as 0, 1, 2 and 3, respectively. The median score of EPVS in BG and CSO were 1 (1, 1) and 2 (1, 3), respectively.

**Figure 2.**
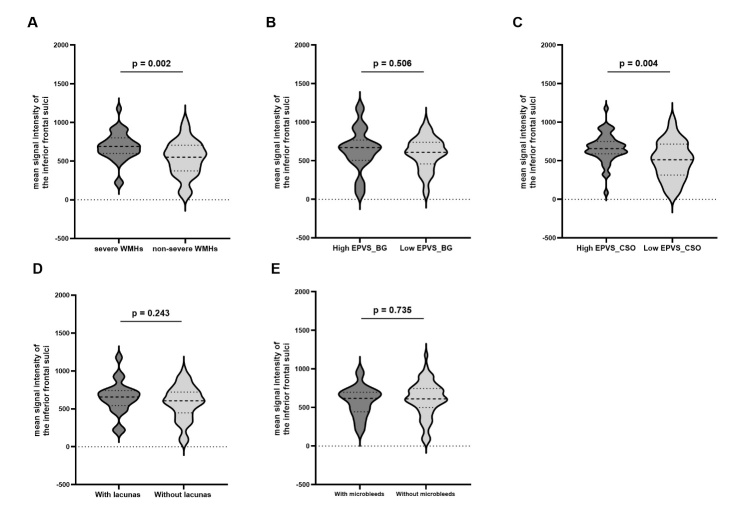
**Association between the mean signal intensity of the inferior frontal sulci and MRI markers of cerebral small vessel disease (CSVD)**. Higher mean signal intensity in the inferior frontal sulci was observed in patients with severe white matter hyperintensities (WMHs) (A) and a higher grade of enlarged perivascular spaces (EPVS) in the centrum semiovale (CSO) (C). However, no significant relationship was found between the mean signal intensity of the inferior frontal sulci and the grade of EPVS in the basal ganglia (BG) (B), lacunas (D) and microbleeds (E). The p-value for independent samples t-tests is displayed in each plot. Each analysis included 76 patients.

**Figure 3.**
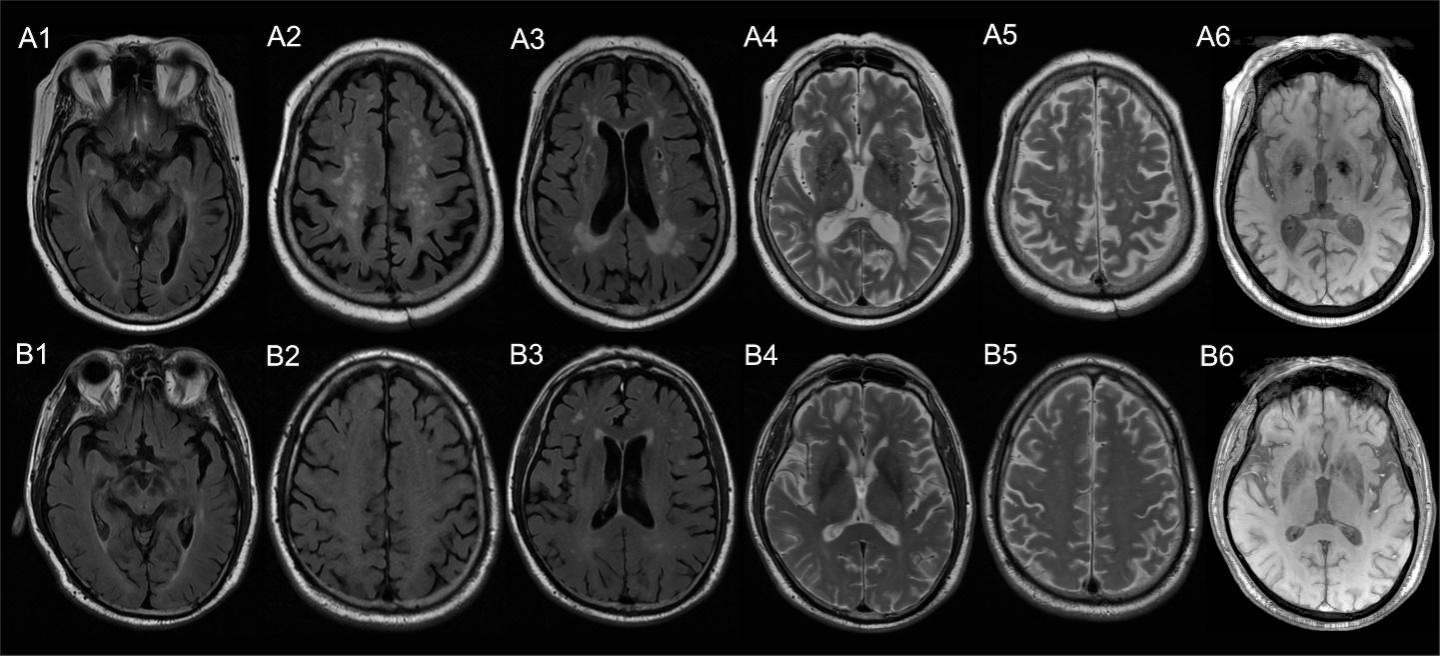
**Representative images of two patients exhibiting different visibility of inferior frontal sulcal hyperintensity (IFSH) and cerebral small vessel disease (CSVD) imaging markers**. Figure A1-A6 were images of a 75-year-old male, with a mean signal intensity of inferior frontal sulci of 657 (A1) and relatively severe CSVD (A2: deep white matter hyperintensities (DWMHs) Fazekas score of 3; A3: periventricular hyperintensities (PVHs) Fazekas score of 3, 1 lacuna; A4: enlarged perivascular spaces (EPVS) in basal ganglia as grade 3; A5: EPVS in centrum semiovale as grade 2; A6:8 microbleeds). Figure B1-B5 were images of a 75-year-old male, with a mean signal intensity of inferior frontal sulci of 412 (B1) and relatively mild CSVD (B2: DWMHs Fazekas score of 2; B3: PVHs Fazekas score of 2, no lacuna; B4: EPVS in basal ganglia as grade 1; B5: EPVS in centrum semiovale as grade 1; B6: no microbleed).

The mean signal intensity of the inferior frontal sulci exhibited a positive correlation with age (r = 0.285, p = 0.012; Pearson Correlation Analysis). However, as shown in Supplementary Table S1, no significant differences were observed in the mean signal intensity of the inferior frontal sulci between patients with and without vascular risk factors, including hypertension, diabetes, hyperlipidemia, and a history of drinking or smoking (all p > 0.05; Independent samples t-tests). As presented in Table 2 and Figure 2, a higher mean signal intensity of the inferior frontal sulci was significantly associated with increased PVHs and DWMHs Fazekas scores (OR 1.0028, 95% CI 1.0006-1.0050; OR 1.0022, 95% CI 1.0001-1.0043; Ordinal logistic regression) and higher grade of EPVS in CSO (OR 1.0033, 95% CI 1.0011-1.0055; Ordinal logistic regression) after adjusting age, sex, hypertension and diabetes. No significant associations were found between the mean intensity of the inferior frontal sulci and other CSVD imaging markers, including the number of cerebral microbleeds, the number of lacunes or the EPVS grade in the BG in the unadjusted or adjusted models (all p > 0.05; Linear or ordinal logistic regression). Figure 3 presented representative images of two patients with varying degrees of IFSH and CSVD imaging markers.

### The relationship between the visibility of IFSH and clearance function of putative mLVs

**Figure 4.**
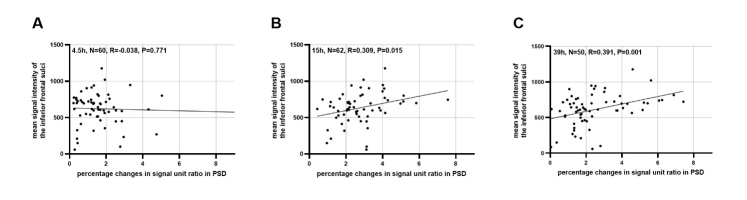
**Relationship between the mean signal intensity of the inferior frontal sulci and percentage change of parasagittal dura (PSD)**. A positive correlation was observed between the mean signal intensity of the inferior frontal sulci and the percentage change in PSD from baseline to 15 hours (n = 62) (B) and 39 hours (n = 50) (C). However, no significant correlation was found between the mean signal intensity of the inferior frontal sulci and the percentage change of PSD from baseline to 4.5 hours (n = 60) (A). Each plot includes information on sample size, the fit line, and the Pearson coefficient (r) with the corresponding p-value.

**Figure 5.**
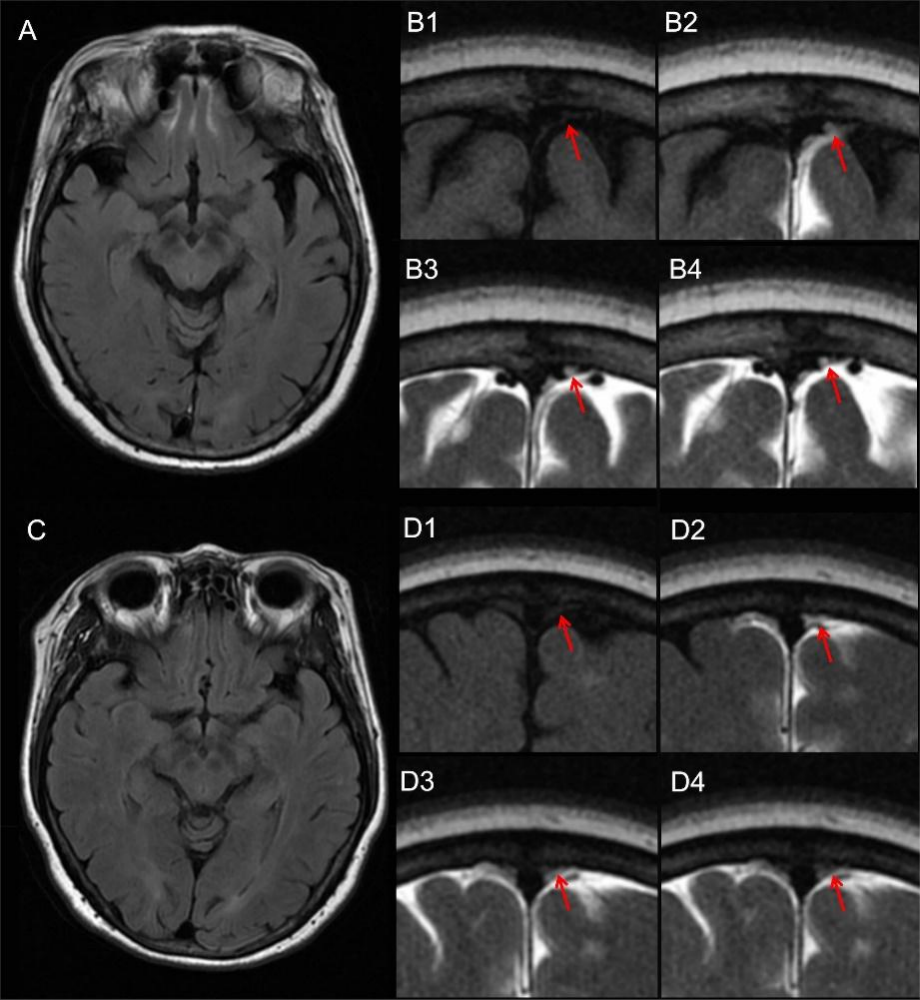
**Representative images of two patients with different visibility of inferior frontal sulcal hyperintensity (IFSH) and clearance function of putative meningeal lymphatic vessels (mLVs)**. Representative images illustrate the putative meningeal lymphatic pathway in two patients with distinct mean signal intensities in the inferior frontal sulci. One patient exhibits a higher mean signal intensity of 1021 (A and B), while another displays a lower mean signal intensity of 609 (C and D). Figures A and C depict axial head 2D Fluid-Attenuated Inversion Recovery (FLAIR) images of the inferior frontal sulci. The clearance of the parasagittal dura (PSD) is depicted in images of coronal head high-resolution FLAIR at different time points: baseline (B1 and D1), 4.5 hours (B2 and D2), 15 hours (B3 and D3), and 39 hours (B4 and D4) following the intrathecal administration of gadodiamide. Red arrows on B and D highlight the altered signal unit ratio in the PSD. Notably, the patient with a higher mean signal intensity of inferior frontal sulci demonstrates higher percentage changes in PSD than the other patient (15 hours: 2.96 vs 2.10; 39 hours: 5.61 vs 1.65).

For the assessment of percentage changes in signal unit ratio in PSD from baseline to 4.5 hours, 15 hours and 39 hours, the inter- and intra-observer consistency had ICC of 0.936, 0.948, 0.929 and 0.916, 0.930, 0.922, respectively. Sixty, 62 and 66 people completed 4.5 hours, 15 hours and 39 hours MRI sessions, respectively. The signal intensity of PSD peaked 4.5, 15, 39 hours after intrathecal administration of a contrast agent in 5 (9.1%), 30 (54.5%) and 20 (36.4%) patients, respectively (Supplementary Table 2). Thirty-five and 20 patients exhibited early and late filling of mLVs, respectively. The average percentage changes of signal unit ratio in PSD from baseline to 4.5 hours, 15 hours and 39 hours were 1.55 ± 1.07, 2.75 ± 1.32, 2.51 ± 1.69, respectively. As demonstrated in Table 3 and Figure 4, a higher mean signal intensity of the inferior frontal sulci was associated with a higher percentage change in PSD from baseline to 15 hours and 39 hours (β = 49.451, p = 0.015; β = 51.999, p = 0.001; Linear Regression Analysis) in the univariate analysis. And this correlation preserved even after adjusting for age, gender, Fazekas of PVHs, Fazekas of DWMHs and Grade of EPVS in CSO (β = 41.149, p = 0.035; β = 53.478, p = 0.001; Linear Regression Analysis). No multicollinearity issues were detected among the covariates across all multivariate models (Supplementary Table 3). Furthermore, the mean signal intensity of the inferior frontal sulci was lower in patients with early filling of mLVs than those with late filling (573.89 ± 201.98 vs 732.70 ± 218.40, p = 0.009; Independent samples t-tests). However, no significant relationships were found between the mean intensity of the inferior frontal sulci and the percentage change of PSD from baseline to 4.5 hours. Figure 5 illustrates representative images of two patients with varying visibility of IFSH and the clearance function of putative mLVs.

### The relationship between the severity of IFSH and cognitive function

Forty-six patients completed the T-MoCA test (Mean = 13.80 (SD = 4.28)), with an average year of education of 7.11 (SD = 4.14). No significant relationships were identified between the mean signal intensity of the inferior frontal sulci and T-MoCA total scores or its subitems (all p > 0.05, Supplementary Table 4). After adjusting for age, gender, education years, and diagnosis of neurodegenerative disease, the mean signal intensity of the inferior frontal sulci remained non-influential on T-MoCA total scores or its subitems (all p > 0.05, Supplementary Table 4).

**Table 2:** Association between the mean signal intensity of the inferior frontal sulci and MRI markers of CSVD.

	Model 1^a^		Model 2^b^		Model 3^c^	
	OR (95% CI)	p value	OR (95% CI)	p value	OR (95% CI)	p value
**Fazekas of PVHs**	1.0034(1.0014-1.0055)	0.001	1.0028(1.0007-1.0050)	0.010	1.0028(1.0006-1.0050)	0.011
**Fazekas of DWMHs**	1.0028(1.0008-1.0048)	0.007	1.0022(1.0000-1.0043)	0.046	1.0022(1.0001-1.0043)	0.042
**Number of lacunas**	1.0009(0.9978-1.0040)	0.576	0.9999(0.9967-1.0032)	0.959	0.9999(0.9967-1.0032)	0.962
**Number of microbleeds**	1.0000(0.9958-1.0043)	0.988	0.9983(0.9940-1.0026)	0.423	0.9981(0.9938-1.0023)	0.370
**Grade of EPVS in BG**	1.0013(0.9989-1.0036)	0.291	0.9989(0.9962-1.0016)	0.418	0.9989(0.9961-1.0016)	0.410
**Grade of EPVS in CSO**	1.0037(1.0016-1.0058)	0.001	1.0033(1.0011-1.0055)	0.003	1.0033(1.0011-1.0055)	0.003

a Model 1 is univariate.

b Adjusted for age, sex.

c Adjusted for age, sex, hypertension and diabetes.

Abbreviation: CSVD, cerebral small vessel disease; PVHs, periventricular hyperintensities; DWMHs, deep white matter hyperintensities; EPVS, enlarged perivascular spaces; BG, basal ganglia; CSO, centrum semiovale.

## DISCUSSION

In this study, we introduced a novel method for assessing the visibility of IFSH by measuring the mean signal intensity of the inferior frontal sulci on 2D FLAIR, offering a practical approach in clinical settings. We evaluated the clearance function of putative mLVs through the percentage changes of signal unite ratio in PSD on DCE-MRI from baseline to 15 and 39 hours following intrathecal administration of a contrast agent. Our investigation established the relationship between the visibility of IFSH and the clearance function of putative mLVs. Additionally, our findings revealed that heightened visibility of IFSH correlated with increasing age, more severe WMHs and a higher grade of EPVS in the centrum semiovale, which suggests a noteworthy association between IFSH and CSVD.

In the current study, the clearance function of putative mLVs was defined as the percentage change of signal unite ratio in the parasagittal dura from baseline to 4.5, 15 and 39 hours after intrathecal injection on dynamic contrast MRI. Previously, an MRI study demonstrated mLVs in healthy subjects following the intravenous injection of gadolinium without disrupting the blood-brain barrier, attributing mLVs enhancement to the leakage of circulating fluids into the CSF [13]. Contrarily, our approach involved the visualization of putative mLVs through direct intrathecal injection of gadodiamide into the CSF. Given gadodiamide's inability to cross the blood-brain barrier, the enhanced tissues along the sagittal sinus are not arteries or veins. The field of view and matrix of 2D-T2-FLAIR in our study are 18 × 18cm^2^ and 320 × 320 pixels, with the resolution stands at 0.5625 × 0.5625 mm^2^ [18]. Human autopsies have identified mLVs ranging from 0.019 to 0.842mm in diameter [13, 19]. Our observations of enhanced signals on 2D-T2-FLAIR post-gadodiamide administration, typically spanning 1 or more pixels, are classified as putative mLVs for several reasons. First, the signals emerge post-gadodiamide, where even sub-pixel tissues can appear enhanced due to high signal intensity. Second, the 3mm slice thickness and oblique mLVs orientations may result in multi-pixel visualizations, contributing to non-sequential appearances. Third, in vivo mLVs, likely larger than collapsed autopsy specimens, expand fully with CSF, which can also explain multi-pixel visualizations in our study. Fourth, the size of putative mLVs identified in our study was similar to the previous study [13].

The correlation between the visibility of IFSH and the clearance function of the putative mLVs lends support to the notion that dysfunction in mLVs contributes to IFSH formation. In our prior study, we noted that the signal intensity of PSD peaked approximately 15 hours after intrathecal administration of a contrast agent in most patients [5]. Consequently, we hypothesized that a higher percentage changes in PSD from baseline to 15 and 39 hours indicated impaired clearance function of mLVs, as the contrast agent in PSD was presumed to flow out of the brain through mLVs after the 15-hour mark. In the current study, we established a positive correlation between the mean signal intensity of the inferior frontal sulci and percentage change of PSD from baseline to 15 hours and 39 hours, providing further support for the involvement of mLVs dysfunction in the formation of IFSH. Abnormal CSF hyperintensity on FLAIR sequences is commonly attributed to an increase of protein, blood or cell debris [2, 3]. Previous research has considered disruptions in blood-brain-barrier or glymphatic dysfunction as potential mechanisms for the leakage of substances into the CSF [1, 20]. When the clearance of mLVs is compromised, the clearance of cerebral macromolecules through the glymphatic pathway is attenuated [7, 21]. Consequently, wastes products, including protein, blood or cell debris, may accumulate in the CSF within the inferior frontal sulci, ultimately leading to the manifestation of IFSH. It is noteworthy that the initial segments of the mLVs are proximate to the inferior frontal sulci in human 3D-rendering of subtraction MRI images [5, 13], providing further support for the hypothesis that the deposition of wastes and proteins in inferior frontal sulci occurs when the clearance function of mLVs is impaired. However, given the inability to obtain CSF samples directly from the inferior frontal sulci, the exact components of IFSH, whether metabolic wastes or proteins, remain unclear. Advanced neuroimaging techniques or histology evidence in future studies are warranted to elucidate the precise components of IFSH.

**Table 3:** Correlations between the mean signal intensity of the inferior frontal sulci and percentage change of PSD.

	Model 1^a^		Model 2^b^		Model 3^c^	
	β (95% CI)	p value	β (95% CI)	p value	β (95% CI)	p value
**Percentage change of PSD from baseline to 4.5 h**	-8.001(-62.679-46.676)	0.771	5.110(-49.506-59.726)	0.852	15.582(-36.044-67.207)	0.548
**Percentage change of PSD from baseline to 15 h**	49.451(10.134-88.769)	0.015	50.861(11.309-90.412)	0.013	41.149(2.928-79.369)	0.035
**Percentage change of PSD from baseline to 39 h**	51.999(21.449-82.550)	0.001	50.819(17.730-83.908)	0.003	53.478(22.030-84.926)	0.001

a Model 1 is univariate.

b Adjusted for age, sex.

c Adjusted for age, sex, Fazekas of PVHs, Fazekas of DWMHs, Grade of EPVS in CSO

Abbreviation: PSD, parasagittal dura; PVHs, periventricular hyperintensities; DWMHs, deep white matter hyperintensities; EPVS, enlarged perivascular spaces; CSO, centrum semiovale.

Our findings indicate a positive association between the severity of IFSH and increasing age, consistent with previous studies, like Zhang et al [1]. Previous research has demonstrated that both the integrity and CSF drainage of mLVs were impaired with aging in rodent models [22]. Studies based on MRI have also shown evidence of impaired mLVs clearance in the aging human brain [5, 23]. Therefore, it can be inferred that, with aging, the accumulation of waste or protein around the CSF drainage pathway near the inferior frontal sulci may lead to the manifestation of IFSH.

The visibility of IFSH in our study was associated with WMH Fazekas scores and EPVS grades in the centrum semiovale, indicating the impact of impaired clearance of mLVs on the development of CSVD [24]. Previous evidence has pointed ischemia and inflammation as crucial pathological processes in CSVD [25, 26]. Impaired clearance of mLVs could lead to the deposition of amyloid protein, increasing the stenosis and occlusion of cerebral microvasculature, thereby exacerbating ischemic and hypoxic reactions and contributing to CSVD [4, 27]. Additionally, impaired drainage via mLVs could worsen the microglial inflammatory response, reducing the clearance of inflammatory cells and cytokines, thereby exaggerating inflammation and CSVD progression [28, 29]. Furthermore, CSVD may reciprocally contribute to glymphatic dysfunction and accelerate the formation of IFSH. Cerebrovascular pulsation is a key driving force for CSF flowing in the glymphatic system [30]. Studies have reported reduced cerebrovascular reactivity and lower CSF stroke volume in patients with a higher burden of WMHs, suggesting that dynamic vascular dysfunction could impede CSF flow in CSVD [31]. Additionally, the perivascular space plays a crucial role in maintaining glymphatic function, and the presence of EPVS may be linked to the failure of interstitial fluid drainage from white matter [18, 32]. This, coupled with amyloid protein deposition in the drainage pathway around the cortex and leptomeningeal arteries, could impair the glymphatic drainage [33].

The significant correlation between the visibility of IFSH and the clearance function of putative mLVs represents a novel and important finding, offering a non-invasive means to assess mLVs clearance function in clinical practice. Since their discovery in 2015, mLVs have been recognized for their crucial role in maintaining brain metabolic and immune balance, and their impairment has been implicated in the pathogenesis of various diseases, including Alzheimer’s disease and traumatic brain injury [21, 30, 34]. However, the study of mLVs in humans is limited, primarily due to the classic evaluation method requiring intrathecal administration of contrast agent and multiple MRI scans [35]. In contrast, the evaluation of IFSH only involves a single 2D FLAIR MRI scan, making it a safer and simpler alternative. Additionally, the visibility of IFSH reflects the instantaneous CSF clearance function at the time of scanning, avoiding the potential influence of sleep during the multiple scans in the classic method. Moreover, the 2D FLIAR sequence used in our study is a widely performed and routine imaging technique in clinical practice, enhancing its potential as a non-invasive biomarker for mLVs in future human studies.

Our research has several limitations. First, the sample size was relatively small, and the complexity of disease components introduced potential bias, especially in the analysis of relationships between the visibility of IFSH and demographic variables and CSVD imaging markers. Secondly, we interpreted signal changes in PSD on MRI scans as indicative of the clearance function of presumed meningeal lymphatic vessels, despite the absence of direct pathological and histological evidence. Future studies providing histological evidence are essential to validate our hypothesis. Third, the assessment of cognitive function was conducted via telephone after discharge, potentially compromising the reliability of cognitive research. Fourth, this is a cross-sectional study. Longitudinal evidence is required to identify the causality between IFSH and the clearance dysfunction of mLVs. Furthermore, the inability to directly obtain CSF samples from the inferior frontal sulci led to a limitation for confirming whether IFSH is caused by the accumulation of waste products. Therefore, further longitudinal studies with larger cohorts and clinical specimens are essential to investigate the mechanisms and clinical significance of IFSH.

In conclusion, our results suggest that the visibility of IFSH assessed on 2D FLAIR may serve as an indicator of the clearance function of putative mLVs, which could be a potential widespread clinical application in the future. Additionally, our study provides evidence supporting that impaired drainage of mLVs could contribute to the development of CSVD.

## Supplementary Materials

The Supplementary data can be found online at: www.aginganddisease.org/EN/10.14336/AD.2024.0415.



## Data Availability

The data that support the fundings of this study are available from the corresponding author, upon reasonable request.
