# Supplementary material for: Inferior Frontal Sulcal Hyperintensity on Fluid-Attenuated Inversion Recovery Is Related to Cerebrospinal Fluid Clearance via Putative Meningeal Lymphatics
Source: Aging Dis. 2024 Apr 15;16(2):1169–79. doi: 10.14336/AD.2024.0415 (PMC11964441; doi:10.14336/AD.2024.0415)
Supplement: Supplementary file 1 [file AD-16-2-1169-s.pdf]

SUPPLEMENTARY DATA

Inferior Frontal Sulcal Hyperintensity on Fluid-Attenuated Inversion Recovery Is Related to Cerebrospinal Fluid Clearance *via* Putative Meningeal Lymphatics

Ziyu Zhou, Ying Zhou, Wang Ran, Shenqiang Yan, Xiao Zhu, Zhongyu Luo, Huihong Ke, Kemeng Zhang, Mengmeng Fang, Jianzhong Sun, Min Lou

© 2024. Zhou Z et al. Published online at http://www.aginganddisease.org/EN/10.14336/AD.2024.0415

SUPPLEMENTARY DATA

Supplemental Method 1. Detailed information about the evaluation of the clearance function of putative meningeal lymphatic vessels (mLVs)

The clearance function of putative mLVs was evaluated by the percentage changes in parasagittal dura (PSD) from baseline to 4.5, 15 and 39 hours after intrathecal administration of contrast agent. Region of interest (ROI) was placed in PSD on high-resolution FLAIR at baseline, 4.5, 15 and 39 hours after intrathecal administration of gadolinium using RadiAnt (Medixant, Poznan, Poland). We recorded the mean signal unit of PSD and normalized it against reference. According to previous studies, we chose vitreous body of the ocular bulb as reference, since there was no significant tracer accumulation in this region after intrathecal injection of gadodiamide [1–3]. For all the time points, the signal unit ratio was derived between PSD and vitreous body of the ocular bulb. The clearance function of putative mLVs was then defined as the percentage change in signal unit ratio of PSD from baseline to 4.5, 15 and 39 hours. The placement of the ROI is illustrated in Supplementary Figure 1.

Supplemental Method 2. Detailed information about the evaluation of cerebral small vessel disease (CSVD) image markers

White matter hyperintensities were defined as white matter with high signal on T2WI and FLAIR and equal or low signal on 3D-T1 (but not the same as CSF signal). According to the Fazekas scale, the WMHs were divided into periventricular hyperintensities (PVHs) and deep white matter hyperintensities (DWMHs). PVHs were graded as absent (score 0), cap (score 1), smooth halo (score 2), or irregular and extending into the subcortical white matter (score 3). DWMHs were graded as absent (score 0), punctate foci (score 1), early-confluent (score 2), or confluent (score 3) [4]. Lacunas were defined on FLAIR as 3 to 15 mm diameter cavities with signal intensities similar to CSF in all performed scan sequences in the whole brain. They were different from enlarged perivascular spaces (EPVS) by size, shape and rim [5]. Microbleeds were defined as small, rounded or circular, well-defined low signal lesions within brain parenchyma with clear margins ranging from 2 to 10 mm in size on susceptibility weighted imaging (SWI) Magnitude images. Signal voids caused by sulci vessels, calcifications, choroid plexus, and low-signal averaging from adjacent bone were excluded [6]. The presence and number of lacunas and microbleeds were recorded. EPVS were defined as small, sharply delineated structures of CSF signal on images that followed the orientation of the perforating vessels and ran perpendicular to the brain surface. EPVS were high signal on T2WI and low signal on 3D-T1 and FLAIR. EPVS in both basal ganglia and centrum semiovale were graded based on as follows: grade 0 (no EPVS), grade 1 (<10 EPVS), grade 2 (11 to 20 EPVS), grade 3 (21 to 40 EPVS), and grade 4 (>40 EPVS) [7].

Supplemental Table 1. The mean signal intensity of the inferior frontal sulci in patients with different demographic information.

	Yes	No	p value
Female	598.65 ± 244.83	607.88 ± 200.36	0.857
Hypertension	651.70 ± 227.29	564.26 ± 211.64	0.087
Diabetes	611.22 ± 197.09	599.06 ± 235.96	0.821
Hyperlipidemia	606.00 ± 200.77	603.12 ± 225.02	0.974
History of drinking	585.39 ± 172.18	608.97 ± 235.95	0.696
History of smoking	583.18 ± 165.83	614.51 ± 248.03	0.559

Supplemental Table 2. The peak time point of signal unit ratio at different locations among all patients.

patient	Peak time point of signal unit ratio at Parasagittal dura
#1	
#2	
#3	
#4	
#5	
#6	
#7	15h
#8	
#9	

© 2024. Zhou Z et al. Published online at http://www.aginganddisease.org/EN/10.14336/AD.2024.0415

SUPPLEMENTARY DATA

**#10**	
**#11**	39h
**#12**	15h
**#13**	15h
**#14**	4.5h
**#15**	15h
**#16**	15h
**#17**	39h
**#18**	15h
**#19**	15h
**#20**	15h
**#21**	15h
**#22**	39h
**#23**	15h
**#24**	39h
**#25**	4.5h
**#26**	39h
**#27**	15h
**#28**	15h
**#29**	15h
**#30**	39h
**#31**	39h
**#32**	39h
**#33**	39h
**#34**	15h
**#35**	39h
**#36**	39h
**#37**	15h
**#38**	39h
**#39**	39h
**#40**	15h
**#41**	15h
**#42**	15h
**#43**	15h
**#44**	15h
**#45**	15h
**#46**	39h
**#47**	
**#48**	15h
**#49**	
**#50**	15h
**#51**	39h
**#52**	15h
**#53**	15h
**#54**	39h
**#55**	
**#56**	
**#57**	
**#58**	4.5h
**#59**	4.5h
**#60**	39h
**#61**	
**#62**	
**#63**	4.5h
**#64**	39h
**#65**	15h
**#66**	
**#67**	15h
**#68**	

© 2024. Zhou Z et al. Published online at http://www.aginganddisease.org/EN/10.14336/AD.2024.0415

SUPPLEMENTARY DATA

**#69**	39h
**#70**	
**#71**	
**#72**	39h
**#73**	15h
**#74**	15h
**#75**	15h
**#76**	

Missing value: the peak time point cannot be determined since the magnetic resonance sequence of the location is not scanned at a certain time point.

Supplemental Table 3. Collinearity analysis in the linear regression model examining the relationship between IFSH and the percentage change of PSD.

	VIF in Model 1[a]	VIF in Model 2[b]	VIF in Model 3[c]
**Percentage change of PSD**	1.088	1.093	1.209
**Age**	1.513	1.403	1.562
**Sex**	1.088	1.080	1.110
**Fazekas of PVHs**	4.019	4.073	4.399
**Fazekas of DWMHs**	3.213	3.048	3.419
**Grade of EPVS in CSO**	1.338	1.334	1.397

[a] Model 1 tested for the relationship between the mean signal intensity of the inferior frontal sulci and the percentage change of PSD from baseline to 4.5h.
[b] Model 2 tested for the relationship between the mean signal intensity of the inferior frontal sulci and the percentage change of PSD from baseline to 15h.
[c] Model 3 tested for the relationship between the mean signal intensity of the inferior frontal sulci and the percentage change of PSD from baseline to 39h.
Abbreviation: IFSH, inferior frontal sulcal hyperintensity; PSD, parasagittal dura; VIF, variance inflation factor; PVHs, periventricular hyperintensities; DWMHs, deep white matter hyperintensities; EPVS, enlarged perivascular spaces; CSO, centrum semiovale.

Supplemental Table 4. Association between the mean signal intensity of the inferior frontal sulci and cognitive function.

	Model 1[a]		Model 2[b]	
	β (95% CI)	p value	β (95% CI)	p value
**T-MoCA total scores**	-0.005 (-0.011- 0.001)	0.124	-0.002 (-0.007- 0.003)	0.430
**Attention and calculation**	-0.002 (-0.004- 0.001)	0.194	-0.001 (-0.004- 0.001)	0.232
**Language**	0.00002 (-0.002- 0.002)	0.975	0.0002 (-0.001- 0.002)	0.775
**Abstraction**	-0.0004 (-0.001- 0.00045)	0.337	-0.0002 (-0.001- 0.001)	0.606
**Delayed recall**	-0.002 (-0.004- 0.001)	0.158	-0.001 (-0.003- 0.002)	0.687
**Orientation**	-0.001(-0.003- 0.001)	0.292	-0.0002 (-0.002- 0.001)	0.777

[a] Model 1 is univariate.
[b] Adjusted for age, sex, education years and diagnosis of neurodegenerative disease.
Abbreviation: T-MoCA, Telephone Montreal Cognitive Assessment.

© 2024. Zhou Z et al. Published online at http://www.aginganddisease.org/EN/10.14336/AD.2024.0415

SUPPLEMENTARY DATA

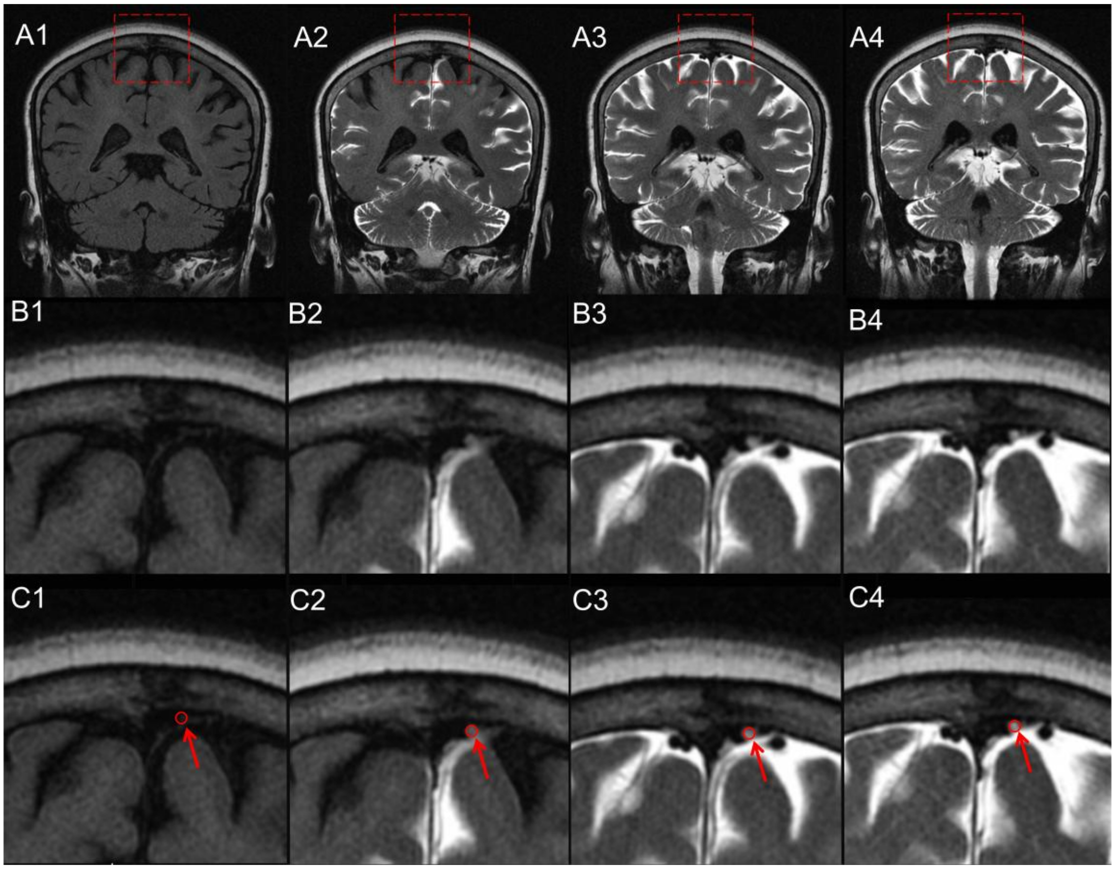

Supplementary Figure 1. Representative images of putative meningeal lymphatic vessels. Panels A, B and C illustrate the visualization of putative meningeal lymphatic pathway on coronal head high-resolution FLAIR images. The red box indicates the position of the parasagittal dura in baseline images (A1), 4.5 hours images (A2), 15 hours images (A3), and 39 hours images (A4) after the intrathecal administration of gadodiamide. Magnified images corresponding to the red box in A1 to A4 are presented in B1 to B4 and C1 to C4. The red circle and red arrow on C1 to C4 highlight the region of interest for evaluating the percentage signal changes in the parasagittal dura (PSD).

References

[1] Eide PK, Vatnehol SAS, Emblem KE, Ringstad G (2018). Magnetic resonance imaging provides evidence of glymphatic drainage from the human brain to cervical lymph nodes. Sci Rep, 8:1–10.

[2] Melin E, Eide PK, Ringstad G (2020). In vivo assessment of cerebrospinal fluid efflux to nasal mucosa in humans. Sci Rep, 10:1–10.

[3] Zhou Y, Cai J, Zhang W, Gong X, Yan S, Zhang K, et al. (2020). Impairment of the Glymphatic Pathway and Putative Meningeal Lymphatic Vessels in the Aging Human. Ann Neurol, 87:357–369.

[4] Fazekas F, Niederkorn K, Schmidt R, Offenbacher H, Homer S, Bertha G, et al. (1988). White matter signal abnormalities in normal individuals: correlation with carotid ultrasonography, cerebral blood flow measurements, and cerebrovascular risk factors. Stroke, 19:1285–1288.

[5] Gouw AA, van der Flier WM, Fazekas F, van Straaten ECW, Pantoni L, Poggesi A, et al. (2008). Progression of white matter hyperintensities and incidence of new lacunes over a 3-year period: the Leukoaraiosis and Disability study. Stroke, 39:1414–1420.

[6] Greenberg SM, Vernooij MW, Cordonnier C, Viswanathan A, Al-Shahi Salman R, Warach S, et al. (2009). Cerebral microbleeds: a guide to detection and interpretation. Lancet Neurol, 8:165–174.

[7] Doubal FN, MacLullich AMJ, Ferguson KJ, Dennis MS, Wardlaw JM (2010). Enlarged perivascular spaces on MRI are a feature of cerebral small vessel disease. Stroke, 41:450–454.

© 2024. Zhou Z et al. Published online at http://www.aginganddisease.org/EN/10.14336/AD.2024.0415
